# Intravenous Injection of PEI-Decorated Iron Oxide Nanoparticles Impacts NF-kappaB Protein Expression in Immunologically Stressed Mice

**DOI:** 10.3390/nano13243166

**Published:** 2023-12-18

**Authors:** Claudia Schwarz, Julia Göring, Cordula Grüttner, Ingrid Hilger

**Affiliations:** 1 Experimental Radiology, Institute of Diagnostic and Interventional Radiology, Jena University Hospital, Friedrich Schiller University Jena, Am Klinikum 1, D-07740 Jena, Germany; claudia.schwarz@krz.uni-jena.de (C.S.); julia.goering@med.uni-jena.de (J.G.); 2 Micromod Partikeltechnologie GmbH, Schillingallee 68, D-18057 Rostock, Germany; gruettner@micromod.de

**Keywords:** polyethyleneimine, iron oxide nanoparticles, NF-kappaB, inflammation

## Abstract

Nanoparticle-based formulations are considered valuable tools for diagnostic and treatment purposes. The surface decoration of nanoparticles with polyethyleneimine (PEI) is often used to enhance their targeting and functional properties. Here, we aimed at addressing the long-term fate *in vivo* and the potential “off-target” effects of PEI decorated iron oxide nanoparticles (PEI-MNPs) in individuals with low-grade and persistent systemic inflammation. For this purpose, we synthesized PEI-MNPs (core–shell method, PEI coating under high pressure homogenization). Further on, we induced a low-grade and persistent inflammation in mice through regular subcutaneous injection of pathogen-associated molecular patterns (PAMPs, from zymosan). PEI-MNPs were injected intravenously. Up to 7 weeks thereafter, the blood parameters were determined via automated fluorescence flow cytometry, animals were euthanized, and the organs analyzed for iron contents (atomic absorption spectrometry) and for expression of NF-κB associated proteins (p65, IκBα, p105/50, p100/52, COX-2, Bcl-2, SDS-PAGE and Western blotting). We observed that the PEI-MNPs had a diameter of 136 nm and a zeta-potential 56.9 mV. After injection in mice, the blood parameters were modified and the iron levels were increased in different organs. Moreover, the liver of animals showed an increased protein expression of canonical NF-κB signaling pathway members early after PEI-MNP application, whereas at the later post-observation time, members of the non-canonical signaling pathway were prominent. We conclude that the synergistic effect between PEI-MNPs and the low-grade and persistent inflammatory state is mainly due to the hepatocytes sensing infection (PAMPs), to immune responses resulting from the intracellular metabolism of the uptaken PEI-MNPs, or to hepatocyte and immune cell communications. Therefore, we suggest a careful assessment of the safety and toxicity of PEI-MNP-based carriers for gene therapy, chemotherapy, and other medical applications not only in healthy individuals but also in those suffering from chronic inflammation.

## 1. Introduction

Many countries are focusing health care systems on their aging population groups. It is expected that patients with several diseases at the same time will increasingly need personalized treatment. Many diseases in the elderly are based on inflammation. Chronic inflammation is a low-grade and persistent inflammatory state and a major risk factor underlying aging and age-related diseases and conditions [1]. Additionally, chronic inflammation can lead to cellular damage, oxidative stress, and dysregulation of immune responses, all of which can contribute to the aging process. While inflammation is a natural response to infections and injuries, a chronic low-level inflammation that occurs in the absence of clinically diagnosed infection can increase the susceptibility to age-related pathologies [2].

To improve health care, researchers and doctors are focusing on the high potential of nanoparticles to allow for controlled drug release, higher drug solubility, bioavailability, and stability. This allows them to use lower doses of combined drugs and therefore reduce the risks of toxicity and side effects for their patients. Moreover, polyethyleneimine (PEI) is a very important polymer, because it exerts distinct adsorption capabilities (e.g., gas, heavy metals, etc.) and its features can be specifically tailored by bonding it with other functional groups (e.g., [3]). Substantial advances have been made using PEI-decorated nanoparticle-based delivery systems. PEI is currently the gold standard, particularly for non-viral gene delivery purposes. The extraordinary cationic charge and buffering capacity of this polymer has been beneficial for complexation with nucleic acids (DNA/RNA) [4]. This complexation process is based on electrostatic interactions of PEI with nucleic acids. The PEI–DNA complexes enter the cells by endocytosis and escape from endosomes by the so-called “proton sponge effect” [5]. Therefore, PEI has further been related to diverse applications such as tissue engineering [6], gene therapy [7], cancer treatment [8], and chemotherapy [9]. 

Furthermore, and to better verify the localization of the PEI-based delivery systems, magnetic nanoparticles (MNPs) made of iron oxide crystals have attracted much attention. The interest of researchers is based on the unique properties of these nanoparticles, such as superparamagnetism, high surface area, large surface-to-volume ratio, and easy separation under external magnetic fields. For these reasons, these nanoparticles offer opportunities for sophisticated magnetic resonance molecular imaging, hyperthermia treatments, or localized drug delivery [10,11,12,13]. Moreover, iron oxide magnetic nanoparticles administered intravenously strongly accumulate in the liver and spleen at 24 h post application [14]. Most other investigations have revealed the biodistribution of PEI-decorated nanoparticles (PEI-MNPs) in laboratory animals at short post observation times after administration only (e.g., [15,16]) and the specific PEI dose was seldom mentioned. There is a lack of knowledge on the long lasting effects after application of iron oxide nanoparticles, which is required in order to improve both their safety and therapeutic potency.

The potential effects of drug (gene) therapy delivery carriers, such as PEI-MNPs, on pro-inflammatory cellular pathways like NF-κB are essential when assessing their safety and efficacy. NF-κB is a signaling cascade that plays a crucial role in regulating the body’s immune and inflammatory responses. It is involved in various cellular processes, including inflammation, immune response, cell survival, and apoptosis. Activation of NF-κB typically occurs in response to infections, inflammation, and cellular stress [17]. 

Current research studies have not explicitly mentioned the specific mechanisms by which PEI-based iron oxide nanoparticles can trigger, or not trigger, inflammation processes. Given the cationic features of PEI, the production of reactive species through the intracellular metabolization of iron (the Fenton reaction, [18]), and finally the liver as the main organ supporting metabolism and immunity, these factors could well impact NF-κB signaling pathways and potentially lead to inflammation. 

In order to unveil the long-term fate *in vivo* and the potential “off-target” effects of nanoparticles with surface-decorated PEI in medical applications, we addressed the health status of mice with chronic inflammation after application of PEI-MNPs. We asked the following questions: (1) How does the presence of a low-grade and persistent systemic inflammation affect the distribution of iron in organs and is there a detectable time dependency? (2) How does the presence of a systemic inflammation influence the impact of the PEI-MNPs on the expression of the NF-κB effector monomers, their inhibitors (IκBα, p105, p100), or the related down-stream proteins in the liver of animals? (3) Does the expression of NF-κB related proteins in the liver of animals change with increasing time after PEI-MNP application? We particularly investigated the relationships after intravenous nanoparticle application, since it has been the favored administration route so far. This offers several advantages in the context of medical applications, particularly in drug delivery and gene therapy. Some of the key advantages include systemic delivery, enhanced circulation time, minimized first-pass metabolism of the cargo, fast onset of action, etc. 

## 2. Materials and Methods

### 2.1. Magnetic Nanoparticles

PEI-MNPs were synthesized by a core–shell method as described previously (e.g., [19,20]). The iron oxide cores were prepared by alkaline precipitation of iron(II) and iron(III) sulfates. The cores were coated with an aqueous solution of PEI (Sigma-Aldrich, 3880, 600–1000 kDa) under high pressure homogenization (see Supplementary Figure S1). The PEI-MNPs were washed with water for injection by magnetic separation and finally filtered (Millex glass fibre filter).

The hydrodynamic diameter and the zeta potential of the PEI-MNPs were measured with a Zetasizer Nano ZS-90 (Malvern Instr. Ltd., Malvern, UK). The zeta potential was measured in a 1 mM KCl solution at a particle concentration of 0.5 mg/mL. The iron concentration was determined with a spectrophotometric method (Spectroquant^®^; Merck) against an iron standard (Titrisol^®^, Merck). Before using the PEI-MNPs in animal trials, their sterility was verified by plating them in agar, incubating them for 72 h at 37 °C, and continuously checking them for the presence of bacterial colonies. All PEI-MNPs were found to be sterile. We used nanoparticles with sizes of between 100 and 150 nm. These are preferred in research and development activities, because of their good circulation half-life and reduced hepatic filtration compared with smaller nanoparticle sizes. 

### 2.2. Animal Studies

The experiments were carried out in accordance with international guidelines on the ethical use of animals, and were approved by the regional animal care committee (Thüringer Landesamt für Verbraucherschutz, Bad Langensalza, Germany, code number: 02-027/15). NMRI female mice (6 to 8 weeks old, Janvier Labs, Saint Berthevin Cedex, France) were treated humanely during the entire experimentation period. They were maintained under artificial day–night cycles (14/20 h light–dark cycles; 21 ± 2 °C room temperature, 55 ± 5% environment humidity) and received food and water ad libitum. Animals were randomly distributed into 4 groups of 3–5 animals each. The so-called “+/+” animal group was the experimental group reflecting the situation in which individuals receive PEI-MNPs in the presence of a low-grade persistent inflammatory state. All other groups were controls: (a) the “−/−” animal group controlled the situation in which both the non- (low-grade and persistent) inflammatory and non-PEI-MNP state were present, (b) the “+/−” animal group that of the low-grade persistent inflammatory state only, and (c) the “−/+” animal group that of the PEI-MNP only state (Table 1). All interventions were carried out with animals under anesthesia with isoflurane (2% (*v*/*v*) in air).

### 2.3. Animal Model for Low-Grade and Persistent Inflammatory State

In order to induce a low-grade and persistent systemic inflammatory state, sterile zymosan (zymosan-A, Sigma-Aldrich Chemie GmbH, Steinheim, Germany, Cat: Z4250) was dissolved in sterile-filtered saline (0.9% (*w*/*v*) NaCl in water) was added to the zymosan. To yield a good mixing, the suspension was vortexed extensively and kept at room temperature overnight and vortexed again just before use. Finally, mice were injected with 18 µg (zymosan per kg body weight), three times sequentially and in cycles of 4 weeks, subcutaneously into the right hind leg of the animals (group “+/+” and “+/−”, Table 1). We chose the mentioned zymosan dose, because higher doses have been shown to induce aggressive inflammation [21], which was not the aim of this study.

**Figure 1:**
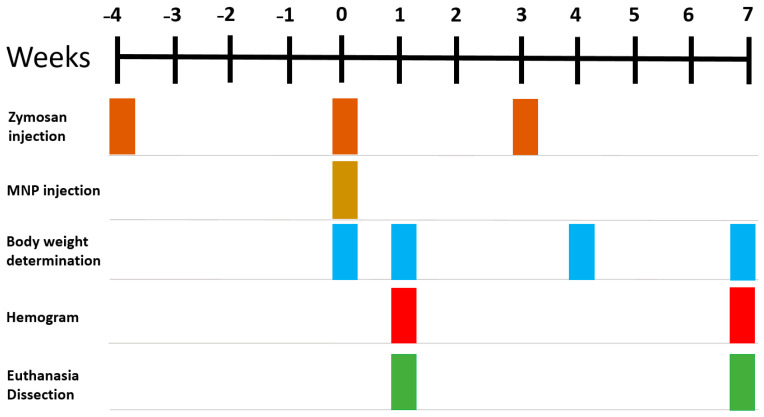
Experimental setup showing the time-dependent interventions into animals during experimentation. Repeated subcutaneous injection of zymosan into the right hind leg (3 cycles of 18 mg/kg body weight each) to induce a low-grade and persistent inflammatory state. MNP: magnetic nanoparticles decorated with polyethyleneimine (PEI), intravenous injection of 50 µmol Fe/kg body weight, 700 µg PEI per mg iron.

Moreover, animal groups “+/+” and “−/+” received PEI-MNPs (50 µmol iron/kg body weight, 700 µg PEI per mg iron, experimental day 0, Figure 1 and Table 1) via the tail vein. The used iron dose was related to the recommendation of the European Medicines Agency for Sinerem^®^, an iron-oxide-based contrast agent for MRI. The amount of PEI per mg iron corresponds to that which is necessary to completely cover (coat) the iron oxide core. Non-injected animals of group “−/−” (Table 1) were used as controls for normal organ iron content and expression of NF-kB associated proteins. We used two post-observation times: (i) 1st week after intravenous application of PEI-MNPs as an early post-observation time, as most other literature data report up to 24 h after PEI-MNP application (e.g., [14,15]), and (ii) 7th week after PEI-MNP application so as to assess the long term effects corresponding to almost 1/10 of the total life expectancy. At said post-observation times (see Figure 1), animals were euthanized with an overdose of isoflurane anesthesia (5% (*v*/*v*)). 

### 2.4. Iron Determination via Atomic Absorption Spectrometry (AAS)

Isolated organ tissues were dried using a hot incubator. Afterwards, 1 mL of a perchloric acid (70%, Cat.: 1005191001, Merck KGaA, Darmstadt, Germany): nitric acid (65%, Cat.: 4989.2, Carl Roth GmbH & Co. KG, 76185 Karlsruhe, Germany) solution (volume proportions 2:3) was administered to dried organs (three pieces per organ and mouse) for incineration. Incineration was performed using a stepwise heating to 70, 160 and 250 °C, holding each temperature for 1 h and cooling down to 22 °C overnight. Afterwards, 1 mL nitric acid (1 N) was added to each tissue sample and the iron content was determined by flame atomic absorption spectrometry (AAS 5 FL, Analytik Jena AG, Jena, Germany) using an acetylene–air flame at the analytical line of 248.3 nm. Aqueous standard solutions were used to calibrate iron contents (0, 5, 10, 20, 30 and 50 μmol Fe per L 0.1 N HCl; Merck KGaA, Darmstadt, Germany). A serial accuracy of 2.4% was determined from 21 dilutions of one tissue sample and a day-to-day accuracy of 4.8% was determined from one sample at 21 consecutive days. 

### 2.5. Detection of Protein Expression via SDS-PAGE and Immunoblotting

Being a frontline immune tissue, the liver was used to detect the expression of proteins associated with the NF-κB signaling pathway. Liver tissue samples from euthanized animals were homogenized in RIPA lysis buffer containing protease and phosphatase inhibitor (both from Roche Diagnostics GmbH, Mannheim, Germany, Cat: 04906845001 and 130-096-427, respectively) using a gentle-MACS™ Octo Dissociator (Miltenyi Biotec B.V. & Co., Bergisch Gladbach, Germany). After centrifugation, the supernatant was taken for determination of protein concentration via Bradford assay using BSA (Carl Roth GmbH & Co. KG, 76185 Karlsruhe, Germany Cat.: 8076.2) as standard (absorption: 595 nm) and a plate reader (Tecan Infinite M1000 Pro, Tecan Group Ltd., Männedorf, Switzerland). Sodium dodecyl sulfate polyacrylamide gel electrophoresis (SDS-PAGE 10% (*w*/*v*) SDS gels) and Western blotting (Immobilon^®^-P, Merck Millipore Ltd., Carrigtwohill, Ireland, Cat.: IPVH00010) were performed to separate and detect proteins in the liver samples. For the immunostaining of separated proteins, membranes were blocked with PUREBlock™ (Vilber Lourmat, Deutschland GmbH, Eberhardzell, Germany, Cat.: PU4010500) and then incubated with primary rabbit antibodies against COX-2, IκBa, Bcl-2 (1:1000 Abcam, Cambridge, UK, Cats: ab179800, ab32518, ab182858, respectively), p100/52, p105/50, (1:1000 Cell Signaling Technology, Leiden, NL, USA, Cats: 52583, 12540), and p65 (1:1000 Santa Cruz Biotechnology, Dallas, TX, USA, Cat.: 8242) overnight at +4 °C. β-actin was used as a protein loading control (1:10,000, Abcam, Cat.: ab20272). Membranes were incubated with a peroxidase-coupled mouse anti-rabbit secondary antibody (1:10,000, Biozol Diagnostica, Eching, Germany; Cat.: 211-035-109, 1 h at room temperature). Chemiluminescence was detected using a specific detector (PURECL, Cat.: PU4100100) and Fusion FX7 Edge (Vilber Lourmat Deutschland GmbH, Eberhardzell, Germany). To quantify the expression of the proteins of the different experimental groups, a densitometric analysis of protein bands was performed using the Bio1D software Version 15.08c (Vilber Lourmat Deutschland GmbH, Eberhardzell, Germany). Data were normalized to β-actin (loading control).

### 2.6. Body Weight and Hemograms

To analyze the impact of PEI-MNPs on the health status of animals, body weight was regularly determined (Figure 1). Further on, the blood of the mice was collected from the subclavian vein of 2.5% (*v*/*v*) isoflurane sedated mice (Figure 1) using a 50 µL anti-coagulant Na–heparin capillary (Hirschmann Laborgeräte GmbH & Co., KG, Eberstadt, Germany). Blood was diluted 1:5 with 200 µL isotonic saline (Fresenius Kabi AG, Bad Homburg, Germany), and finally measured on an automated hematology analyzer for animals (Sysmex XT-1800i, Hyogo, Japan).

### 2.7. Statistical Analysis

Data were plotted as mean ± standard deviation of the mean. To determine the statistical significance between experimental groups, *t*-test with Welch’s correction or Mann–Whitney U test was used and differences between the animal groups were considered statistically significant when *p* < 0.05.

## 3. Results

The PEI-MNPs were found to contain a distinct amount of iron, a hydrodynamic diameter of 136 nm and a positive surface charge as result of their decoration with PEI (Table 2 and Supplementary Figure S2). The iron weight fraction in the nanoparticles was 80% (*w*/*w*). 

**Figure 2:**
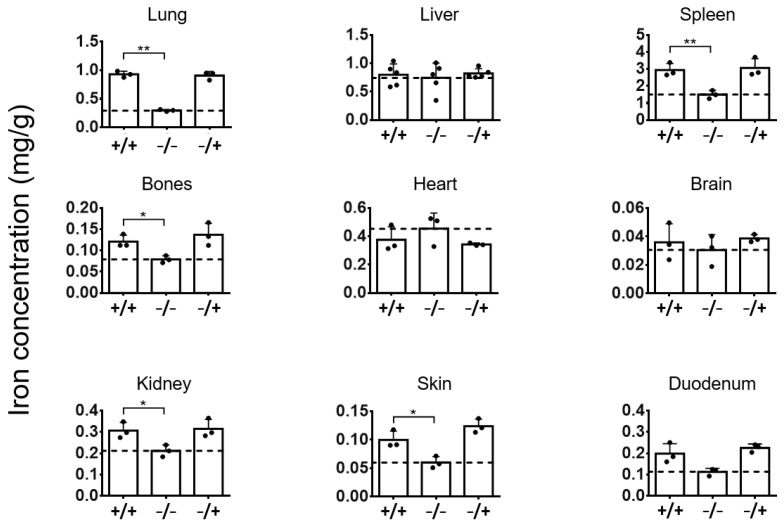
Iron concentrations in organs of animals with low-grade persistent inflammatory state at one (1) week after intravenous application of PEI-MNPs. Experimental groups: “+/+”: animals with low-grade persistent inflammation state (subcutaneous injection of zymosan, 3 times 18 µg/kg body weight) and with intravenous injection of PEI-MNPs (50 µmol Fe/kg body weight, 700 µg PEI per mg iron); “−/−”: animals without low-grade persistent inflammation state and without intravenous injection of PEI-MNPs; “−/+”: animals without low-grade persistent inflammation state but with intravenous injection with PEI-MNP. Data is plotted as mg iron per g dry tissue mass, and as mean and standard deviation of the mean, *n* = 3 to 5 animals per group, * *p* < 0.05, ** *p* < 0.01 (*t*-test with Welch’s correction).

The iron determination in tissues demonstrated that the administration of PEI-MNPs to animals with a low-grade and persistent inflammatory state (animal group “+/+”) caused an increase of iron-levels in the lung, spleen, bones, brain, kidneys, skin, and duodenum in comparison with the non-diseased and non-MNP administered animals (group “−/−”, post observation time: 1 week after PEI-MNP administration, Figure 1 and Figure 2). Interestingly, the mentioned increase of iron content was comparable to the PEI-MNP administered only group (no inflammatory state, animal group “−/+”), indicating that the detected organ iron concentration resulted from the PEI-MNP administration per se. Furthermore, the iron contents in the liver of the animal group “+/+” were almost insignificant, as the corresponding values were comparable to the “−/−” and “−/+” animal groups. In the heart, the administration of PEI-MNPs in the animal group “+/+” reduced the iron content by tendency while at the same time being comparable to the “−/+” animal group.

**Figure 3:**
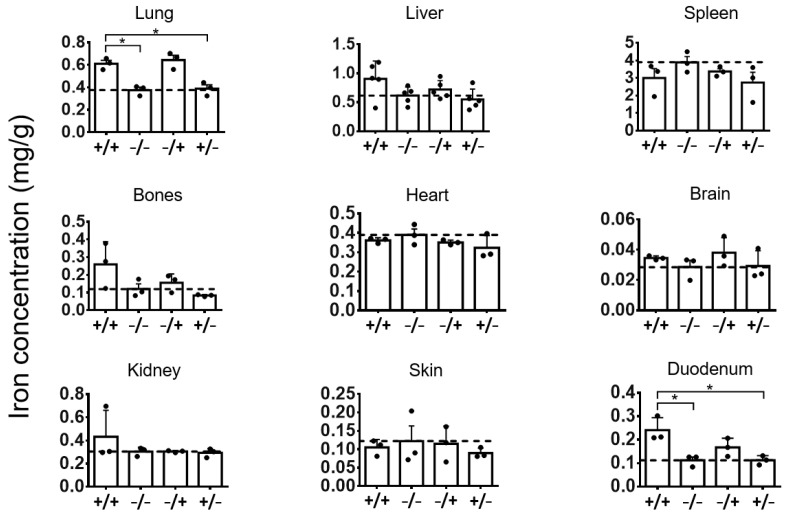
Iron concentrations in the organs of animals with low-grade persistent inflammatory state at seven (7) weeks after intravenous application of PEI-MNPs. Experimental groups: “+/+”: animals with low-grade persistent inflammation state (subcutaneous injection of zymosan, 3 times 18 µg/kg body weight) and with intravenous injection of PEI-MNPs (50 µmol Fe/kg body weight, 700 µg PEI per mg iron); “−/−”: animals without low-grade persistent inflammation state and without intravenous injection of PEI-MNPs, “−/+”: animals without low-grade persistent inflammation state but with intravenous injection of PEI-MNP, “+/−”: animals with low-grade persistent inflammation state but without intravenous injection of PEI-MNP. Data are plotted as mg iron per g dry tissue mass, and as mean and standard deviation of the mean, *n* = 3 to 5 animals per group, * *p* < 0.05 (*t*-test with Welch’s correction).

With increasing post observation times (7 weeks after PEI-MNP administration, Figure 1 and Figure 3), the comparatively increased iron organ levels (see above) persisted particularly in the lung and duodenum of the animal group “+/+”, which again is attributed to the presence of the PEI-MNPs per se (almost comparable levels with the “−/+” animal group). Furthermore, the liver and bones of the animal group “+/+” showed, by tendency, increased iron contents compared with those of the control animal groups. Interestingly, the organ iron levels of the “+/−” animal group were almost inconspicuous or, by tendency, lower when compared with the “−/−” animal group (liver, spleen, bones, heart, and skin, Figure 3).

**Figure 4:**
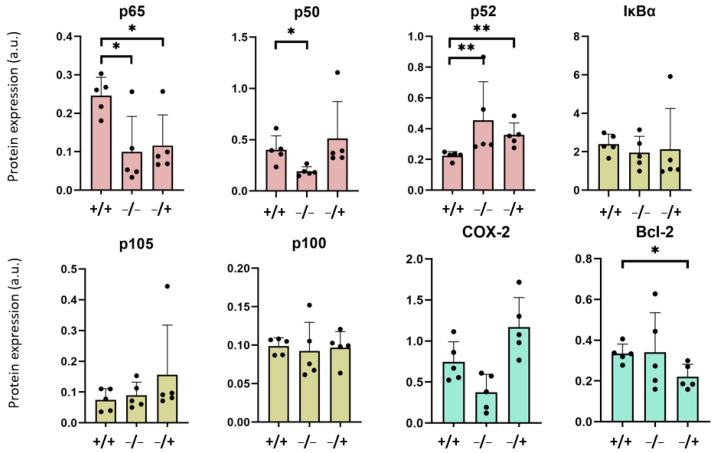
Protein expression of the important players of the NF-κB signaling pathway in the liver of animals with low-grade persistent inflammatory state after one (1) week after intravenous application of PEI-MNPs. Experimental groups: “+/+”: animals with low-grade persistent inflammation state (subcutaneous injection of zymosan, 3 times 18 µg/kg body weight) and with intravenous injection of PEI-MNPs (50 µmol Fe/kg body weight, 700 µg PEI per mg iron); “−/−”: animals without low-grade persistent inflammation state and without intravenous injection of PEI-MNPs; “−/+”: animals without low-grade persistent inflammation state but with intravenous injection of PEI-MNP; “+/−”: animals with low-grade persistent inflammation state but without intravenous injection of PEI-MNP. Protein expression normalized to the housekeeping cellular protein β-actin. NF-κB nuclear factors in red, regulators in green and effector proteins in blue. Data are plotted as mean and standard deviation of the mean, *n* = 5 animals per group, * *p* < 0.05, ** *p* < 0.01 (Mann–Whitney U test).

The analysis of expression of NF-κB monomers at the early post observation time (1 week after PEI-MNP administration, Figure 4) revealed an overexpression of p65 and p50 NF-κB nuclear factors in the livers of the “+/+” animal group, compared with those of the “−/−” and “−/+” groups. Both proteins are responsible for gene activation after acute immune stimuli (canonical pathway, [17]). At the same time, the expression of the p52 NF-κB nuclear factor (non-canonical pathway, mainly associated with persistent immune responses, [22]), was reduced in the livers of the “+/+” animal group (compared with the “−/−” and “−/+” animal groups, Figure 4). Interestingly, the protein expressions of the NF-κB signaling pathway regulator IκBα, p105, and p100 of the “+/+” animal group were not distinctly different from those of the other groups, showing that neither the low-grade and persistent inflammation, the presence of PEI-MNP, nor any synergistic effects between them played a significant role in the liver (Figure 4). Looking at defined downstream proteins of the NF-κB signaling pathway, there was a tendency for an increased COX-2 expression in the liver of the “+/+” animal group, which was mainly due to the presence of the PEI-MNPs per se (protein expression almost as high as in the “−/+” animal group, but higher than the “−/−” animal group, Figure 4). 

**Figure 5:**
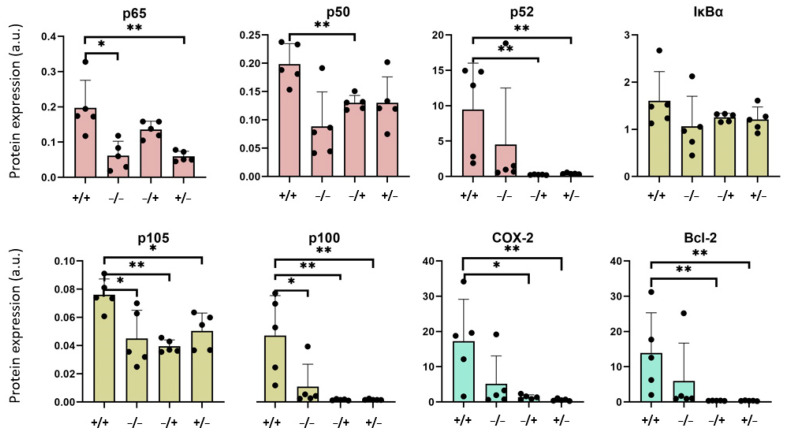
Increased protein expression of important players of the NF-κB pathway in the liver of animals with low-grade persistent inflammatory state at 7 weeks after intravenously injected PEI-MNP. Experimental groups: “+/+”: animals with low-grade persistent inflammation state (subcutaneous injection of zymosan, 3 times 18 µg/kg body weight) and with intravenous injection of PEI-MNPs (50 µmol Fe/kg body weight, 700 µg PEI per mg iron); “−/−”: animals without low-grade persistent inflammation state and without intravenous injection of PEI-MNPs; “−/+”: animals without low-grade persistent inflammation state but with intravenous injection of PEI-MNP. Protein expression normalized to the housekeeping cellular protein β-actin. NF-κB nuclear factors in red, regulators in green and effector proteins in blue. Data are plotted as mean and standard deviation of the mean, *n* = 5 animals per group. * *p* < 0.05, ** *p* < 0.01 (Mann–Whitney U test).

With increasing post observation times (7 weeks after PEI-MNP administration), the NF-κB nuclear factors p65 and p50 in the liver of the “+/+” animal group were still distinctly overexpressed, but at this timepoint the non-canonical nuclear factor p52 was also prominent (compared with the “−/−” and “−/+” animal groups, Figure 5). At the same time the NF-κB expression in livers of the inflammation only animal group (“+/−”) were almost inconspicuous when compared with the other animal groups. Additionally, the regulators of the NF-κB signaling pathway IκBα, p105 and p100, were overexpressed in the “+/+” animal group, which is attributed to the synergistic effects between the low-grade and persistent inflammatory state and the presence of the PEI-MNPs (expression of NK-κB regulators was relatively low in all other animal groups, Figure 5). The same relationships between the different animal groups apply for the expression of the two downstream effector proteins COX-2 and Bcl-2 (Figure 5). 

Furthermore, it is of note that when comparing the protein expression in the liver of the “−/+” and “+/−” animal groups, most players of the NF-κB signaling pathway showed an almost comparable expression (Figure 4 and Figure 5). 

Additionally, when plotting the protein expression of the different NF-κB nuclear protein players in dependence on time, a decreased expression of members of the canonical pathway, at least by tendency, and an increased expression of those of the non-canonical pathway can be appreciated in the liver of the “+/+” animal group (Supplementary Figure S3). Interestingly, this is also valid for the non-inflammatory and non-MNP administered (“−/−”) animal group and the PEI-MNP only animal group, at least by tendency (“−/+”, Supplementary Figure S3). Finally, there was a time-dependent increased expression of COX-2 and Bcl-2 in all animal groups (Supplementary Figure S4). 

**Figure 6:**
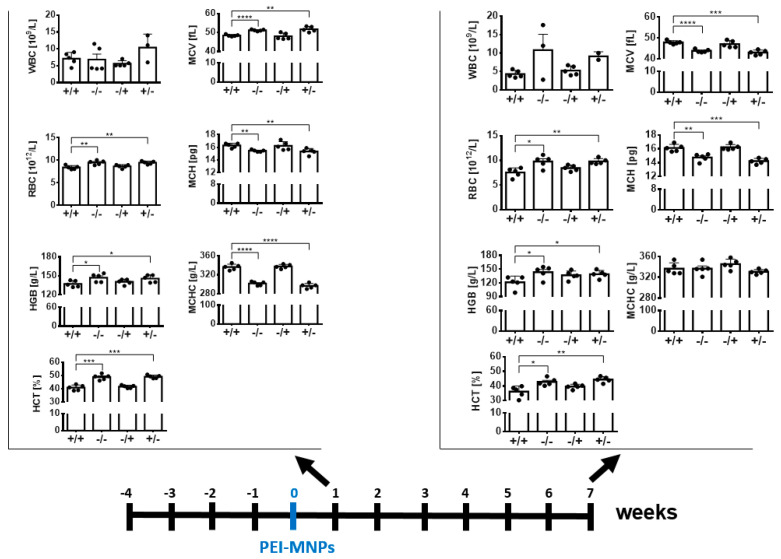
Hemogram of animals with low-grade persistent inflammatory state in comparison with controls after intravenous application of PEI-MNPs. Experimental groups: “+/+”: animals with low-grade persistent inflammation state (subcutaneous injection of zymosan, 3 times 18 µg/kg body weight) and with intravenous injection of PEI-MNPs (50 µmol Fe/kg body weight, 700 µg PEI per mg iron; “−/−”: animals without low-grade persistent inflammation state and without intravenous injection of PEI-MNPs; “−/+”: animals without low-grade persistent inflammation state but with intravenous injection of PEI-MNP. WBC: white blood cells, RBC: red blood cells, HGB: hemoglobin, HCT: hematocrit MCV: red blood cell mean volume, MCH: mean red blood cell hemoglobin, MCHC: mean cell hemoglobin concentration. Data are plotted as mean and standard deviation of the mean, *n* = 5 animals per group. * *p* < 0.05, ** *p* < 0.01, *** *p* < 0.001, **** *p* < 0.0001 (unpaired *t*-test with Welch’s correction).

The hemograms of the “+/+” animal group showed reduced values for hemoglobin (HGB), hematocrit (HCT), and the number of red blood cells (RBC), whereas the average amount of hemoglobin per red blood cell (MCH) and the red blood cell mean volume (MCV) were increased in the same animals (when compared with the “−/−” animal group, Figure 6). Additionally, the “+/+” animal group showed signs of reduced white blood cell count (WBC, leukopenia; compared with the “−/−” animal group, Figure 6). Interestingly, the blood parameters of the “+/+” animal group mostly resembled those of the “−/+” group. 

Finally, the macroscopic analysis of the organs of all animal groups revealed no morphological abnormalities at both post observation times (Supplementary Figure S5). Additionally, the body weight of animals in each of the groups was unchanged (Supplementary Figure S6).

## 4. Discussion

In general, the intravenous application of PEI-MNPs to animals with low-grade and persistent inflammation induced the following effects: (i) it changed the iron levels in different organs in a time-dependent manner, (ii) it increased the expression of members of the canonical NF-κB signaling pathway soon after PEI-MNP application and later also that of the non-canonical signaling pathway in the liver of the animals, and (iii) it induced leukopenia and alterations of the red-blood cell parameters. 

Our PEI-MNPs are nanocomposites [23], and they showed the physicochemical features which have been extensively reported in a separate publication [24] compiled in the frame of the same joint research project (nanoBEL, German Federal Ministry of Education and Research). In the mentioned publication, the PEI-MNPs are shown to be stable over several months in appropriate storage conditions (sterile water, 4 °C). Further on, they degrade in a stimulated endo-lysosomal environment within 72 h (determined by FT-IR, differential scanning calorimetry, etc.), whereas they are almost stable in normal intercellular fluids (pH 7.4 at 37 °C) over 28 days. Accordingly, the degradation process is the result of amine protonation, attraction of anions and water, shell separation and finally degradation of the iron oxide core.

In this study, the particular analyses of the iron contents give information on the localization of the PEI-MNPs and/or the intracellular degradation and metabolization of the MNPs after intravenous injection in laboratory animals. Once in the blood, the PEI-MNPs are opsonized and consequently easily recognized by tissue-resident macrophages in highly vascularized organs, such as the lung, liver, spleen, bones, etc. [25]. Tissue-resident macrophages (e.g., alveolar macrophages, Kupffer cells, etc.) then remove them from circulation by phagocytosis and degrade/metabolize them in endolysosomes [26]. The fact that the iron content in the organs of the “+/+” animal group were as high as those from animals receiving PEI-MNPs only (“−/+” group) well reflects the mentioned relationships. 

Furthermore, the high iron levels in the tissue of the excretory organs, like the kidneys, skin, and duodenum, from animals with a low-grade and persistent inflammatory state and that were administered PEI-MNP (“+/+” animal group) demonstrate specific iron elimination processes at the early post observation time. Such processes seem to take place independently of the low-grade and persistent systemic inflammatory state (“+/+” animal group compared with the “−/+” one). This also indicates that important iron storage activities are present in the bone marrow and spleen (1 week after administration). 

At later post-observation times, distinctly high iron concentrations were found in the lung, bones, liver, and kidneys of the “+/+” animal group. All of these are organs that consist of large amounts of tissue-resident macrophages located in reticular connective tissues. Because these phagocytic cells readily take up nanoparticles in general and cationic PEI-MNPs in particular [27], we expect that nanoparticle degradation and metabolization (see below) is still taking place in those cells. At the same time, increased amounts of iron appeared in the duodenum of the “+/+” animal group compared with the “−/−” and the “+/−” animal groups. Because the “−/+” control animal group did not show any increased iron levels, we attribute the mentioned effects in the “+/+” animal group to the combined influence of the systemic inflammation and iron excretion. 

In general, the liver of animals showed a distinct expression pattern of NF-κB protein players. Notably, amine groups of the PEI-MNP coating can easily form hydrogen bonds with hydroxyl groups of cells, contributing to its high adhesion and absorption properties [28]. Additionally, there seems to be a hierarchy of nanoparticle uptake that is seen among liver cells, which is characterized by the dominance of Kupffer cells, followed by liver sinusoidal endothelial cells, hepatic stellate cells and limited access to hepatocytes [29]. Furthermore, hepatocytes comprise the majority (~85%) of the liver mass and more recent studies have emphasized a role for hepatocytes as active drivers in liver inflammation through intercellular communication. This means that hepatocytes are involved in the production of so-called acute phase proteins after receiving pathogenic and inflammatory signals from immune cells (e.g., cytokines like IL-6, IL-22, IL-1β and TNF-α, [30]), in our study this applied to immune cells in organs with increased iron amount (e.g., lung, spleen, kidneys, and duodenum, see above). Hepatocytes respond to immune cells by secreting innate immunity proteins to the bloodstream, such as opsonins, PAMP-signaling regulators, iron-metabolism-related proteins etc. [30]. This reveals that hepatocytes constitutively produce and secrete a variety of mediators that play important roles in immune regulation [31]. Therefore, we generally attribute the increase of nuclear factor proteins NF-κB in the liver of the “+/+” animal group to the major contribution of hepatocytes in the inflammatory response. 

Additionally, we relate the increased expression of NF-κB protein players in the liver of the “+/+” animal group to the fact that the animals were experiencing two synergistic stress stimuli. In particular, immune cells are continuously activated due to the presence of zymosan (i.e., binding of “PAMP receptors” like toll-like receptors (TLRs), and pattern recognition receptors) and due to PEI-MNP uptake by same immune cells (e.g., by macropinocytosis and clathrin-mediated endocytosis [32,33]). In the end, said synergistic effects obviously influenced the intracellular NF-κB-signaling pathway protein expression, as our results show. In agreement with our findings, it has been observed that PEI per se has a strong immune activity [34] and that PEI can cause nuclear translocation of p50 and p65 subunits of NF-κB, suggesting the roles of PEI in the stimulation of NF-κB signaling [35]. 

Additionally tissue-resident macrophages as well as hepatocytes of animals of the “+/+” group could well have been damaged by the production of hydroxyl radicals (the Fenton reaction, [36]) after uptake and intracellular metabolization of PEI-MNPs, which caused oxidative stress and overexpression of several NF-κB proteins in the liver. Namely, during endosomal nanoparticle degradation, free Fe^2+^ molecules are released to the labile iron pool in the cytosol or to mitochondria [37].

Intriguingly, the impact of the sole application of PEI-MNPs on the expression of NF-κB protein players in the liver is only weak at least for the late post-observation time (comparison of the “−/+” and “−/−” animal groups). One explanation is that in absence of persistent PAMPs from zymosan, the immune stressors emerging from the metabolization of PEI-MNPs are not sufficient to induce a distinct overexpression of the NF-κB protein players on their own. Another explanation is that important protein overexpression may have taken place unnoticed, since we used only two time points after nanoparticle administration (1 and 7 weeks). In this view, previous research studies show that there is a high PEI-nanoparticle accumulation in the liver as early as 24 h after intravenous administration [14,15,16].

The presence of the low-grade inflammation state only slightly affected the NF-κB protein expression, as consequence of the regular administration of PAMPs using regular zymosan injection. The exact mechanism by which zymosan, a yeast glucan, induces systemic inflammation is less well known than that of other PAMPs, such as the lipopolysaccharides from the membranes of Gram-negative bacteria (LPS), which are sensed by TLR4-positive cells. TLR4 activation triggers NK-κB activation and downstream inflammatory responses in target cells. Interestingly, this activation is strongly regulated by LPS-binding proteins mainly produced by hepatocytes (e.g., [38]). This means that hepatocytes are involved in sensing the presence of PAMPs by immune and epithelial cells. NF-κB is an important transcription factor controlling this process [39]. We postulate that similar relationships do also apply for PAMPs emerging from zymosan and that the slight effects on NF-κB protein expression observed in the liver of the “+/−” animal group is caused, at least in part, to the sensing of zymosan-derived PAMPs by hepatocytes. 

The protein regulators p105 and p100 of the non-canonical NF-κB signaling pathway were distinctly overexpressed in the liver of the “+/+” animal group, particularly at the late post-observation time. Because the non-canonical NF-κB signaling pathway requires processing to p100, it is quite specialized for a particular intracellular scenario. Consequently, its activation requires time, but its activation is more persistent than that of the canonical pathway [22,40]. 

Interestingly, the two exemplary pro-inflammatory downstream proteins COX-2 and Bcl-2 were significantly overexpressed in the “+/+” animal group in comparison with the PEI-MNP only or the low-grade and persistent inflammation only (PAMPs) group. Again, this reflects the situation that both stressors, the PEI-MNPs and the zymosan, act synergistically when activating COX-2 and Blc-2, via involvement of the NF-κB signaling pathway. It is of note that animals administrated with PEI-MNP only showed a transient high COX-2 protein expression in the liver (during the early stages of observation, when compared with the other animal groups). This observation could well be related to pro-inflammatory effects during nanoparticle degradation (see above). Notably, all animal groups also experienced a certain time-dependent increase of COX-2 and Bcl-2 expression in the liver. However, since the mentioned levels are comparable with those of the “−/−” animal group, this is the result of normal age-dependent metabolic, immune, and detoxification processes in the liver. 

Moreover, the hemogram of the “+/+” animal group was striking in relation to that of the control groups. In particular, the changed values for HGB, HTC, RBC, MCH, MCV, and MCHC indicate that the body is preventing from using stored iron to make enough healthy blood cells (e.g., [41,42]). Additionally, leukopenia was observed in these animals, particularly at the late post-observation time. Because the blood parameters of the “+/+” animal group were mostly similar to those of the “−/+” animal group, we suggest that the presence of the PEI-MNPs, per se, causes changes in the systemic iron metabolism and consumes white blood cells as a consequence of the their pro-inflammatory insult (e.g., [43,44]). 

**Figure 7:**
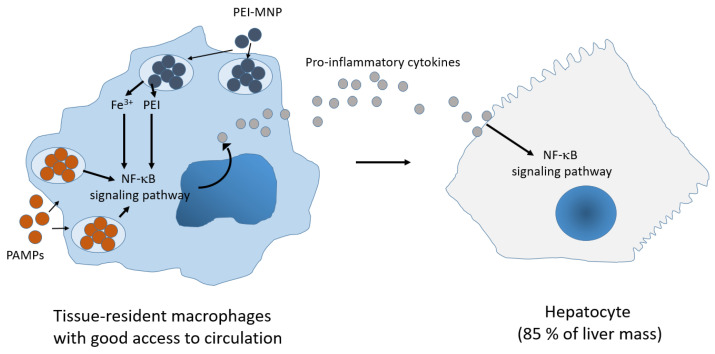
Proposed mechanism of the mutual effect of PEI-MNPs on both the tissue-resident macrophage hepatocytes during a systemic low-grade and persistent inflammatory state. Hepatocytes sense the pro-inflammatory cytokines from tissue-resident macrophages after uptake of PEI-MNPs.

In general, we have shown that the intravenous injection of PEI-MNPs in animals with a low-grade and persistent inflammation state is able to influence the NF-κB signaling pathway in the liver in a specific manner (Figure 7). The described changes in protein expression are known to occur as a consequence of modifications in the protein turnover, which take place when a protein is increasingly “consumed” through specific metabolic processes. For example, the regulator of the canonical NF-κB signaling pathway IκBα, when phosphorylated (activated), is degraded in an ubiquitin-dependent process [45]. Therefore, we assume that the observed changes in expression of the NF-κB signaling pathway protein players are related, at least indirectly, to mechanisms of NF-κB activation. 

We expect that a similar scenario could take place in immunologically stressed patients, particularly those with multi-morbidities. Further research studies should reveal how signaling cascades other than NF-κB affect the liver metabolism as consequence of the intravenous injection of PEI-MNP in individuals with low-grade and persistent inflammation. Of particular interest are pathways involved in the regulation and production of pro-inflammatory gene expression and cytokine production, like the mitogen-activated protein kinase (MAPK) pathway, the Janus kinase-signal transducer and activator of transcription (JAK-STAT) pathway, etc. 

To further complete the understanding of the long-term *in vivo* nanoparticle fate described here, additional studies should also focus on the impact of PEI-MNPs on cytokine levels in blood. In particular, cytokines released by lymphocytes are crucial when modulating the immune response, co-stimulating other immune cells, and producing antibodies but also in suppressing immune responses, with the latter particularly contributing to the persistence of chronic inflammation. Because low-grade inflammation in aging individuals is associated with the dysregulation of immune function, of cytokine secretion, and of signaling molecule regulation in immune responses, animals of several age groups should be considered as well. Finally, the most important chronic diseases are triggered by distinct underlying mechanisms which can affect different tissues and organs, whereas the specific inflammatory mediators among these can vary. For that reason, the impact of PEI-MNPs should be further elucidated using different chronic inflammation disease models.

Finally, when synthesizing nanoparticles for a defined therapy, the observations described here should encourage researchers to carefully occupy the free amine groups of the PEI-MNPs with the corresponding target molecules (negative nucleic acids, acidic proteins, etc.). This procedure will allow for the effective control of their fate in the body as well as the potential “off-target” effects of PEI-MNPs, which may counteract with the intended ones.

## 5. Conclusions

Our data reveal that the intravenous administration of PEI-MNPs to animals with low-grade and persistent inflammation altered the iron levels of different organs and the expression of important players of the NF-κB signaling pathway. The presence of PEI-MNPs, per se, induce changes in iron metabolism and turnover of white blood cells. We postulate the induction of synergistic effects between PEI-MNPs and the low-grade and persistent inflammatory state in the liver. These are due, at least in part, to the sensing of the hepatocytes of PAMPs during infection, to immune responses resulting from the intracellular metabolism of the uptaken PEI-MNPs, and/or to hepatocyte and immune cell communications. Among the most important players of the NF-κB signaling pathway, canonical members were overexpressed earlier and the non-canonical member at later observation times. As the impact of PEI-MNP alone or the low-grade and persistent inflammatory state alone on the expression of important NF-κB protein players was rather inconspicuous, we suggest a careful assessment of the safety and toxicity of PEI-MNP-based carriers for gene therapy, chemotherapy, and other medical applications, not only in healthy individuals but also in those suffering from chronic inflammation.

## Figures and Tables

**Table 1:** Overview of animal groups used in the present study. Animals were euthanized at post-observation times 1 and 7 weeks. Note that for technical reasons, the animal group “−/+” was used for the post-observation time of 7 weeks only.

Name	Systemic Inflammation	Injected PEI-NPs
+/+	yes	yes
−/−	no	no
−/+	no	yes
+/−	yes	no

**Table 2:** Physico-chemical features of the PEI-NPs. C: concentration, NP: nanoparticles, Fe: iron, HD: hydrodynamic diameter, ZP: zeta potential.

C(NP) (mg/mL)	C(Fe) (mg/mL)	HD (nm)	ZP (mV)
25	15.6	136	56.9

## Data Availability

Data will be available upon request.

## Supplementary Materials

The following supporting information can be downloaded at: https://www.mdpi.com/article/10.3390/nano13243166/s1, Figure S1: Schematic view on the synthesis of PEI-MNPs; Figure S2: Morphological features of PEI-MNPs. Figure S3: Time dependent decrease of protein players of the canonical and increase non-canonical NF-κB pathway in the liver of animals with low-grade persistent inflammatory state. Figure S4: Time-dependent expression of typical downstream proteins of the NF-κB pathway in the liver of animals with low-grade persistent inflammatory state. Figure S5: Light images of extracted organs from animals with low-grade persistent inflammatory state in comparison to controls at seven (7) weeks after intravenous application of PEI-MNPs. Figure S6: Body weight of animals with low-grade persistent inflammatory state after intravenous application of PEI-MNPs in comparison to controls..
