# Supplementary material for: Intravenous Injection of PEI-Decorated Iron Oxide Nanoparticles Impacts NF-kappaB Protein Expression in Immunologically Stressed Mice
Source: Nanomaterials (Basel). 2023 Dec 18;13(24):3166. doi: 10.3390/nano13243166 (PMC10745731; doi:10.3390/nano13243166)
Supplement: Supplementary file 1 [file nanomaterials-13-03166-s001.zip › nanomaterials-2744020-supplementary.pdf]

nanomaterials

MDPI

Supplementary materials

Intravenous Injection of PEI-Decorated Iron Oxide Nanoparticles Impacts NF-Kappa B Protein Expression in Immunologically Stressed Mice

Claudia Schwarz [1], Julia Göring [1], Cordula Grüttner [2] and Ingrid Hilger [2,*]

[1] Experimental Radiology, Institute of Diagnostic and Interventional Radiology, Jena University Hospital, Friedrich Schiller University Jena, Am Klinikum 1, D-07740 Jena, Germany; claudia.schwarz@krz.uni-jena.de (C.S.); julia.goering@med.uni-jena.de (J.G.)

[2] Micromod Partikeltechnologie GmbH, Schillingallee 68, D-18057 Rostock, Germany; gruettner@micromod.de

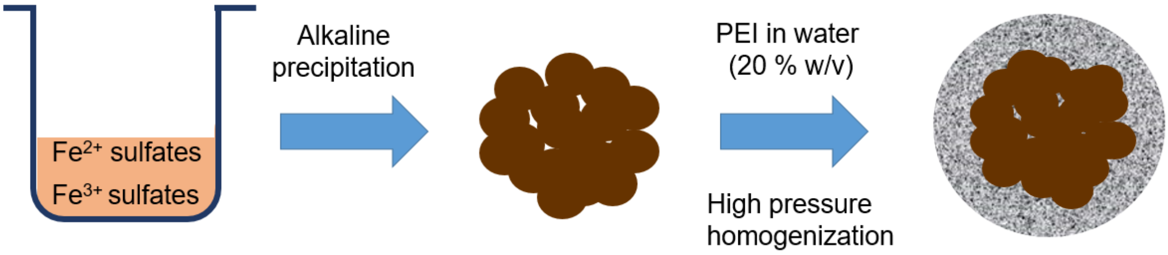

Supplementary Figure S1. Schematic view on the synthesis of PEI-MNPs.

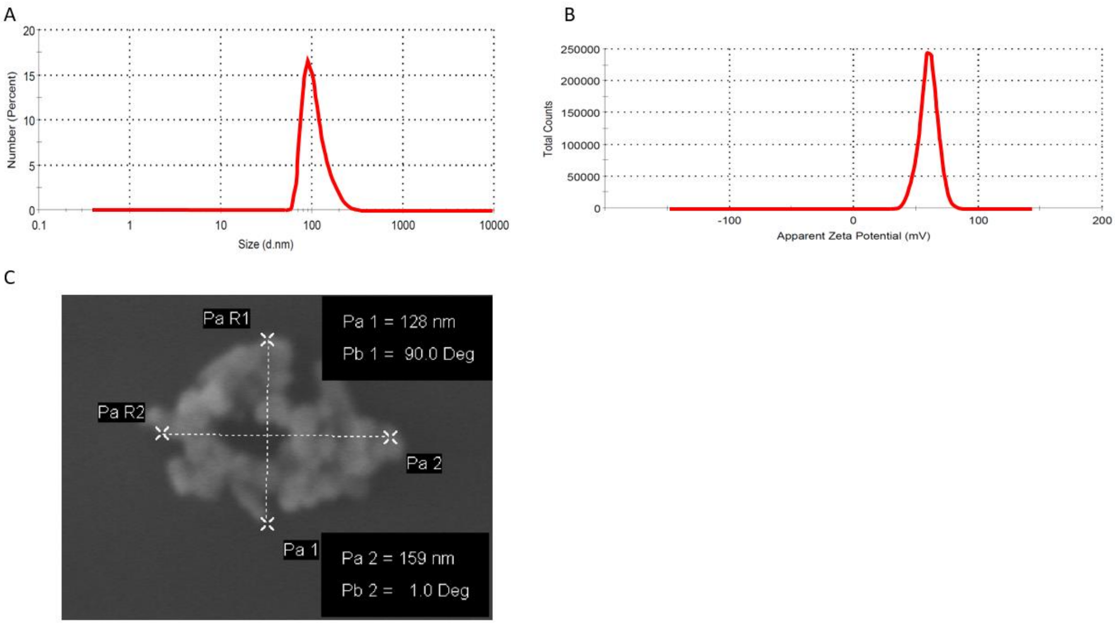

Supplementary Figure S2. Morphological features of PEI-MNPs. A) Size distribution by number, B) Zeta potential distribution, c) SEM image (magnification: 157 kx, electron high tension: 5 kV, working distance: 8 mm, aperture size: 20 μm).

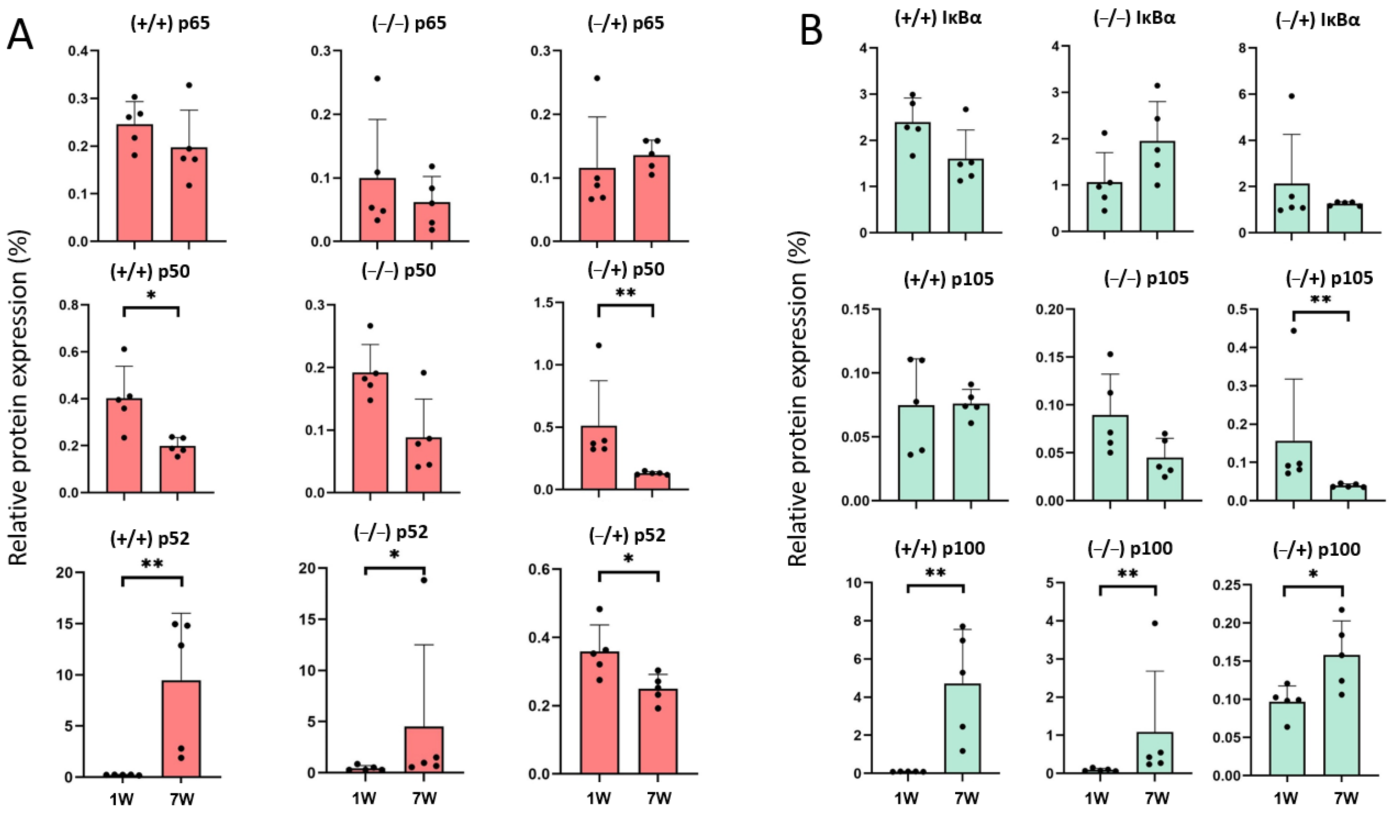

Supplementary Figure S3: Time dependent decrease of protein players of the canonical and increase non-canonical NF-κB pathway in the liver of animals with low-grade persistent inflammatory state. A) NF-κB nuclear factors and B) NF-κB regulators. Experimental groups: "+/+" : animals with low-grade persistent inflammation state (subcutaneous injection of zymosan, 3 times 18 µg/kg body weight) and with intravenous injection of PEI-MNPs (50 µmol Fe/kg body weight, 700 µg PEI per mg iron); "-/-": animals without low-grade persistent inflammation state and without intravenous injection of PEI-MNPs, "-/+": animals without low-grade persistent inflammation state but with intravenous injection of PEI-MNP. Protein expression was normalized to the housekeeping cellular protein β-actin. Data is plotted as mean and standard deviation of the mean, n = 5 animals per group. *p < 0.05 **p < 0.01 (Mann-Whitney U test); W: week

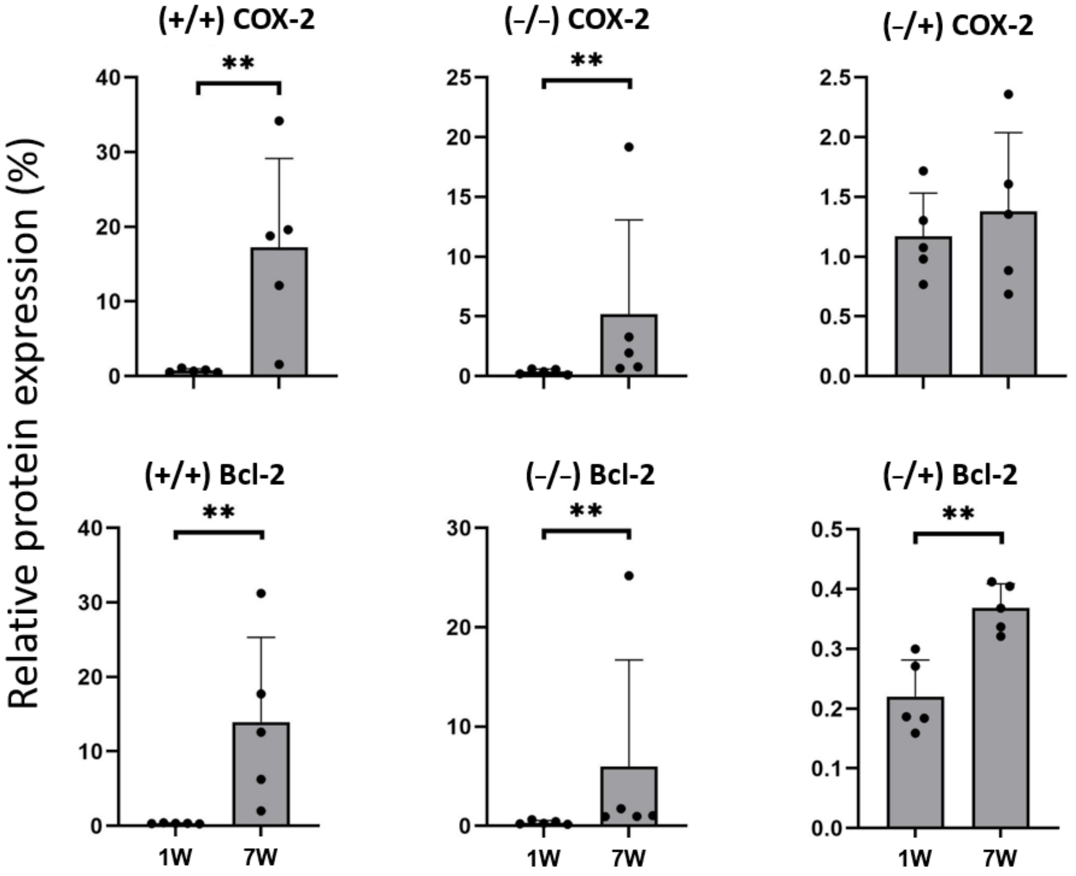

Supplementary Figure S4. Time-dependent expression of typical downstream proteins of the NF-κB pathway in the liver of animals with low-grade persistent inflammatory state. Experimental groups: "+/+" : animals with low-grade persistent inflammation state (subcutaneous injection of zymosan, 3 times 18 μg/kg body weight) and with intravenous injection of PEI-MNPs (50 μmol Fe/kg body weight, 700 μg PEI per mg iron); "-/-": animals without low-grade persistent inflammation state and without intravenous injection of PEI-MNPs, "-/+": animals without low-grade persistent inflammation state but with intravenous injection of PEI-MNP, "+/-": animals with low-grade persistent inflammation state but without intravenous injection of PEI-MNP. Protein expression normalized to the housekeeping cellular protein β-actin. Data is plotted as mean and standard deviation of the mean, n = 5 animals per group, $p < 0.01$ (Mann-Whitney U test), W: week.

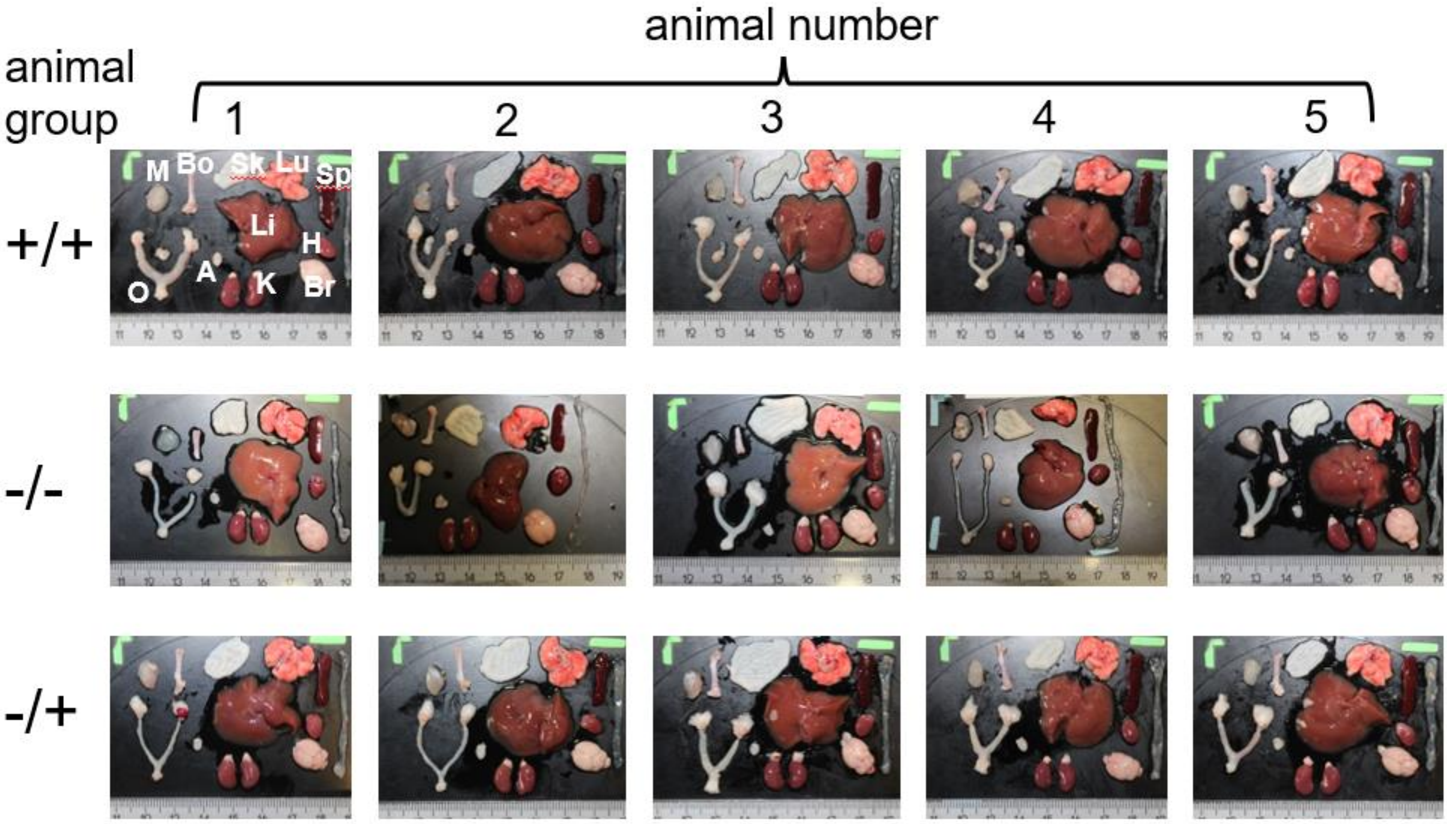

Supplementary Figure S5. Light images of extracted organs from animals with low-grade persistent inflammatory state in comparison to controls at seven (7) weeks after intravenous application of PEI-MNPs. Experimental groups: "+/+" : animals with low-grade persistent inflammation state (subcutaneous injection of zymosan, 3 times 18 µg/kg body weight) and with intravenous injection of PEI-MNPs (50 µmol Fe/kg body weight, 700 µg PEI per mg iron); "-/-": animals without low-grade persistent inflammation state and without intravenous injection of PEI-MNPs, "-/+": animals without low-grade persistent inflammation state but with intravenous injection of PEI-MNP. M: muscle, Bo: Bones, Sk: skin, Lu: lungs, Sp: spleen, H: heart, Br: brain, Li: liver, K: kidney, A: adrenal glands, O: ovary and uterus.

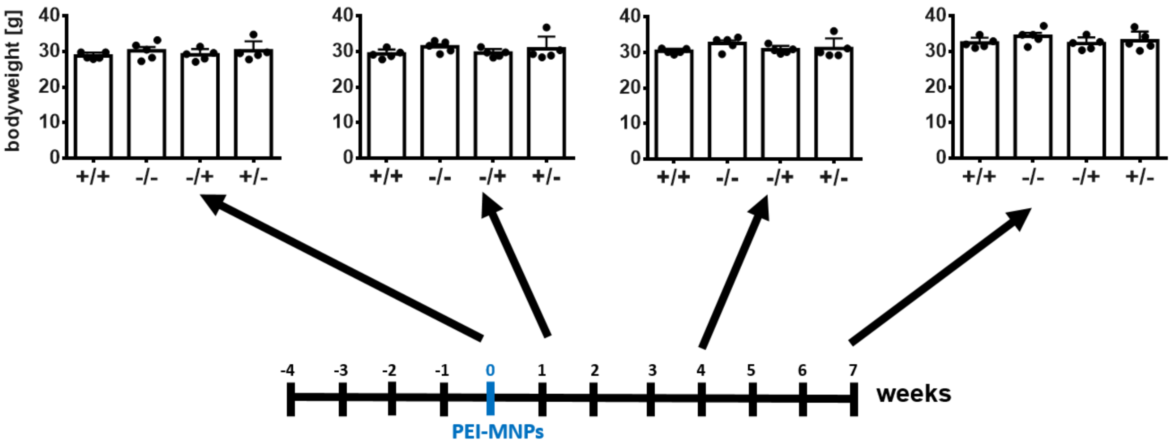

Supplementary Figure S6. Body weight of animals with low-grade persistent inflammatory state after intravenous application of PEI-MNPs in comparison to controls. Experimental groups: "+/+" : animals with low-grade persistent inflammation state (subcutaneous injection of zymosan, 3 times 18 µg/kg body weight) and with intravenous injection of PEI-MNPs (50 µmol Fe/kg body weight, 700 µg PEI per mg iron)"-/-": animals without low-grade persistent inflammation state and without intravenous injection of PEI-MNPs, "-/+": animals without low-grade persistent inflammation state but with intravenous injection of PEI-MNP. Data is plotted as mean and standard deviation of the mean, n = 5 animals per group.
